# Chelsea Hospital for Women

**Published:** 1894-07-28

**Authors:** 


### The Chelsea Hospital for Women.

### Resignation of the Medical Staff.

At a meeting of the Chelsea Vestry, Mr. Wright moved that a vote of thanks be given to Lord Cadogan and the committee of investigation into the working of the Chelsea Hospital, and that the vestry considered that the action of their medical officer was fully justified. Mr. C. P. Doll seconded the resolution, which was adopted. Mr. Irons moved that the committee's report should be sent to the Home Secretary, and to the President of the Local Government Board, and that the former should be invited to institute an inquiry into the deaths that had taken place. This was carried, as was a further resolution requesting the Registrar-General to appoint medical officers of health as superintendent registrars in order to avoid any doubt about the *bona fides* of death certificates.

A special meeting of the governors was held on the 24th inst., under the chairmanship of the Earl of Cadogan. His lordship said that the committee were competent to make the inquiry which resulted in the report. It was the duty of the board of management to accept the conclusions of that committee, and he should not be prepared to join in any fresh inquiry or in any argument upon the conclusions arrived at. The board of management had resolved to carry out all the recommendations in the report; but they had received letters from the entire medical staff resigning their positions in the hospital, at the same time denying that they had been guilty of any improper treatment of patients, and expressing their willingness to continue their work until some other arrangements could be made. The board of management had resolved to accept these resignations.

Lord Cadogan read a letter signed by Dr. W. H. Fenton, Dr. Vincent Dickinson, Dr. Frank F. Schacht, Dr. A. D. Leith Napier, Dr. J. A. Shaw-Mackenzie, Dr. A. E. Giles, and Dr. T. Watts Eden, and the signatories stated that they felt it incumbent to hand in their resignations. Dr. W. Travers had written that in order to enable the board to take such steps as it considered best he resigned, but added, "I am unconscious of having in any way failed in the faithful discharge of my duties during the eleven years that I have held the appointment." Dr. Fancourt Barnes, who also wrote resigning, pointed with satisfaction to the fact that the mortality after operations of all kinds that he had performed was only 3·3 per cent.

His lordship added that there was no mystery or concealment about the report. As a result of the inquiry and the report, he mentioned that on the previous day he received a report of the Hospital Sunday Fund, in which it was stated that this hospital had been excluded from any benefit "consequent upon the very unsatisfactory condition of its wards, and the public complaints made against the professional staff and general management." Reconstruction, under such circumstances, was absolutely required.

